# Apo AIV and Citrulline Plasma Concentrations in Short Bowel Syndrome Patients: The Influence of Short Bowel Anatomy

**DOI:** 10.1371/journal.pone.0163762

**Published:** 2016-09-30

**Authors:** M. Dolores López-Tejero, Núria Virgili, Jordi Targarona, Jorge Ruiz, Natalia García, Denise Oró, Judit García-Villoria, Gloria Creus, Ana M. Pita

**Affiliations:** 1 Departament de Bioquimica i Biomedicina Molecular, Facultat de Biologia, Universitat de Barcelona, Barcelona, Spain; 2 Unitat de Nutrició i Dietètica, Servei d’Endocrinologia i Nutrició, Hospital Universitari de Bellvitge (HUB), IDIBELL, L’Hospitalet de Llobregat, Barcelona, Spain; 3 MIX*e*STAT S.L., Barcelona, Spain; 4 Sección de Errores Congénitos del Metabolismo-IBC, Servicio de Bioquímica y Genética Molecular, Hospital Clínic de Barcelona, CIBERER, IDIBAPS, Barcelona, Spain; University Hospital Llandough, UNITED KINGDOM

## Abstract

**Introduction:**

Parenteral nutrition (PN) dependence in short bowel syndrome (SBS) patients is linked to the functionality of the remnant small bowel (RSB). Patients may wean off PN following a period of intestinal adaptation that restores this functionality. Currently, plasma citrulline is the standard biomarker for monitoring intestinal functionality and adaptation. However, available studies reveal that the relationship the biomarker with the length and function of the RSB is arguable. Thus, having additional biomarkers would improve pointing out PN weaning.

**Aim:**

By measuring concomitant changes in citrulline and the novel biomarker apolipoprotein AIV (Apo AIV), as well as taking into account the anatomy of the RSB, this exploratory study aims to a better understanding of the intestinal adaptation process and characterization of the SBS patients under PN.

**Methods:**

Thirty four adult SBS patients were selected and assigned to adapted (aSBS) and non-adapted (nSBS) groups after reconstructive surgeries. Remaining jejunum and ileum lengths were recorded. The aSBS patients were either on an oral diet (ORAL group), those with intestinal insufficiency, or on oral and home parenteral nutrition (HPN group), those with chronic intestinal failure. Apo AIV and citrulline were analyzed in plasma samples after overnight fasting. An exploratory ROC analysis using citrulline as gold standard was performed.

**Results:**

Biomarkers, Apo AIV and citrulline showed a significant correlation with RSBL in aSBS patients. In jejuno-ileocolic patients, only Apo AIV correlated with RSBL (r_b_ = 0.54) and with ileum length (r_b_ = 0.84). In patients without ileum neither biomarker showed any correlation with RSBL. ROC analysis indicated the Apo AIV cut-off value to be 4.6 mg /100 mL for differentiating between the aSBS HPN and ORAL groups.

**Conclusions:**

Therefore, in addition to citrulline, Apo AIV can be set as a biomarker to monitor intestinal adaptation in SBS patients. As short bowel anatomy is shown to influence citrulline and Apo AIV plasma values, both biomarkers complement each other furnishing a new insight to manage PN dependence.

## Introduction

Short bowel syndrome (SBS) is a clinical condition that includes a reduction of the enterocyte mass, which is a consequence of the removal of a large amount of anatomical and functional intestine. This implies a severe malabsorption condition in which parenteral nutrition (PN) dependence can be either chronic or transient [1].

PN dependence is significantly associated with the remnant small bowel length (RSBL), which is chronic or long term for patients with a RSBL <50 cm and transient for patients with a RSBL ranging between 50 cm and 150 cm [2, 3]. Influenced by the presence of colon, PN weaning is linked to the restoration of patients’ enteral food tolerance and nutritional autonomy. This is achieved at the end of the intestinal adaptation process that may last, on average, up to two years [4–7]. Over this period, the intestinal mucosa grows, the villi thicken and the remnant bowel recovers some of the lost functionalities and mass [6].

Exploring the potential of non-invasive plasma biomarkers of intestinal function can be clinically useful to assess and monitor the condition of SBS patients [8–11]. Citrulline and apolipoprotein AIV (Apo AIV) are both candidates to fulfill this role because their concentrations in plasma mainly depend on production in the small intestine and they are not affected from liver uptake (first-pass metabolism). Citrulline is a non-protein amino acid almost exclusively produced by enterocytes as by-product of glutamine metabolism [12]. It is converted into arginine by the kidney [13]. Apo AIV in humans is exclusively synthesized by enterocytes [14]. It is very abundant as it accounts for up to 4% of the proteins synthesized by enterocytes. Apo AIV is incorporated into the surface of nascent chylomicrons. Upon entering the blood circulation, it is rapidly dissociated from the chylomicrons and predominates in the plasma as lipoprotein-free fraction [15]. In humans, it shows no circadian rhythm [16] and maintains stable physiological plasma levels under a regular oral regimen [17].

Hitherto, citrulline has been widely used to monitor the state of patients with extensive enterocyte loss in SBS, Crohn’s disease, radiation and chemotherapy enteritis [18, 19]. In contrast, Apo AIV has been used in fewer clinical studies on intestinal mass and functionality [20–23]. Thereby, Apo AIV’s potential as a biomarker remains relatively untested. The main advantages of Apo AIV over citrulline are that it requires simple equipment, a small sample and generates quicker results.

Citrulline concentrations were found to correlate with RSBL [18, 24] enterocyte mass and function [25, 26] However, Peters et al. [27] noted that the high correlation found by Luo et al. [24] failed if the cohort of three patients with >300 cm RSBL were treated as outliers. The gold standard of citrulline as a biomarker was attested to by the threshold values, 20 μM in adult patients [18, 19] and 15 μM in children [9], for distinguishing between chronic and transient PN patients, which have been confirmed by several authors [3, 24]. However, in a few patients under PN care, some studies show citrulline values higher than the thresholds [1, 9, 18]. Conversely, citrulline values lower than thresholds are found in PN weaned patients [9, 18]. Therefore, since citrulline is not totally reliable, the decision to wean PN still depends on a set of subjective and objective variables: patient reports, serial blood and urine analysis and patient’s overall physical condition [8, 24].

One factor that may influence biomarkers’ values in SBS patients is the anatomy of the remnant small intestine after surgery. According to function and structure, the small intestine can be divided into a proximal part, i.e., duodenum and jejunum, and a distal part, i.e., the ileum. The main differences between the proximal and distal parts are length, luminal surface, absorptive capacity, synthesis of amino acids and expression of proteins. For instance, while citrulline is mainly synthesized in the proximal part [10, 28]. Apo AIV is expressed in the ileum, which, in turn, stimulates production in the jejunum [16]. Hence, bowel adaptation may involve sections, such as the ileum, which are scarcely active in the biosynthesis of citrulline [19]. Therefore, citrulline concentrations alone may not be sufficient to monitor intestinal adaptation in SBS patients. The anatomy of the RSBL and additional biomarkers should also be considered.

The aim of this research was to study the potential Apo AIV as a novel biomarker and the influence of the RSBL anatomy in the values of Apo AIV and citrulline to achieve greater insight into the process of intestinal adaptation and PN dependence.

## Materials and Methods

### Patients

Adult stable SBS patients of European descent (n = 34, 19 women and 15 men, aged between 26 and 83 years) were recruited in the Nutrition Unit from the HUB (Barcelona, Spain). Ethnicity, age and gender-matched healthy subjects were enrolled as a control group (n = 39, 19 women and 20 men). Following Jeppesen et al. [29], SBS patients were classified into adapted SBS (aSBS; n = 26) and non-adapted SBS (nSBS; n = 8) groups. The inclusion criterion for adapted SBS patients was a minimum period after surgical resection of 18 months. Samples from patients were taken between 20 months and 228 months after re-establishment of the digestive circuit. Patients within the aSBS group were split into two categories: aSBS ORAL (n = 19) for those with intestinal insufficiency, which were only on an oral diet and aSBS HPN (n = 7) for those with chronic intestinal failure, which had an oral diet and home parenteral nutrition (HPN) (Table 1 and S1 Table). These categories correspond with the ESPEN definition of intestinal failure in adults [30].

**Table 1:** Descriptive characteristics of all patients.

Patient characteristics	Summary statistics	aSBS (adapted)		nSBS
		HPN	ORAL	(non-adapted)
		(n = 7)	(n = 19)	(n = 8)
**Age (years)**	Mean (SD)	44.7 (16.5)	60.1 (14.4)	52.5 (12.7)
	Median	43	64	54
**Sex (n)**	Men (%)	4 (57)	7 (37)	4 (50)
	Women(%)	3 (43)	12 (63)	4 (50)
**Weight (kg)**	Mean (SD)	57.8 (12.3)	63.3 (15.3)	59.2 (15.1)
	Median	58	60	57
**Remnant Small Bowel Length RSBL (cm)**	Mean (SD)	25.7 (14.0)	118.4 (40.7)	109.4 (83.9)
	Median	20	100	75
**Anastomosis type (n)**	Type I	0	3	6
	Type II	4	9	0
	Type III	3	7	2
**Total energy intake (MJ/ day)**	Mean (SD)	15.2 (2.3)	10.9 (2.2)	11.0 (0.8)
	Median	15.5	11.0	10.5
**Oral energy intake (MJ / day)**	Mean (SD)	11.8 (2.2)	10.9 (2.2)	9.6 (1.9)
	Median	11.5	11	10
**Parenteral energy intake (MJ/day)**	Mean (SD)	3.5 (0.9)	0	3.3 (0.8)
	Median	3.9	0	3.2 (n = 4)

In the nSBS group, intestinal resection was performed between 0 and 3 months before enrollment, and included patients with intestinal failure Type II [30] (n = 8) still in their intestinal adaptation stage. Before intestinal reconstruction (<3 months) only four patients of this group received solely an oral diet, the remainder (n = 4) received complementary HPN (Table 1 and S1 Table).

In three of nSBS patients, the process of their intestinal adaptation was closely monitored: patient ‘1’, a 43-year-old male had a jejunocolic anastomosis with 60 cm jejunum (RSBL) and reported hyperphagia, well beyond the period of adaptation, a minimum of 5 years after the resection; patient ‘2’, a 44-year-old male had jejunoileocolic anastomosis, 70 cm RSBL (36% ileum); and patient ‘3’, a 48-year-old female also had jejunoileocolic anastomosis, 150 cm RSBL (33% ileum). Apo AIV and citrulline concentrations were measured in their plasma at distinct time periods, i.e., “baseline” stage at <3 months before intestinal reconstruction and at two adaptation time periods following re-establishment of the digestive circuit: “12 to 15 months” which is considered an initial state of stability and “5 years” which is considered a time for total adaptation [4, 7]. After the baseline moment, patients only received an oral diet.

Exclusion criteria were: active infection, liver or renal failure or both, metabolic diseases, HIV-positive status and active neoplasia. Patients were normolipidemic; they received neither steroids nor immunomodulators. Etiology of intestinal resection in selected SBS patients was of ischemic (59%) or actinic origin (41%). RSBL was taken from the surgeon’s report. Depending on the extent of resection in SBS, digestive circuit anastomoses may be classified into (Table 1 and S1 Table): Type I (jejunostomy), only remnant jejunum (n = 9); Type II (jejunocolic), remnant jejunum and partial or total colon (n = 13); and Type III (jejunoileocolic), reduced jejunum and ileum with partial or total colon (n = 12).

This study was approved by the ethics committee at the Hospital Universitari Bellvitge (HUB, Barcelona, Spain). Approval was recorded under file reference number 133/04. All SBS patients gave written consent. Control patients were healthy volunteers that gave verbal consent for their inclusion, as the hospital norms do not require their written consent for participation.

### Nutritional management

Both, adapted and non-adapted SBS patients showed no signs of malnutrition with mean BMI (Kg/m^2^) values within the normal range (aSBS HPN: 20.9; aSBS ORAL: 23 and nSBS: 21,8). Table 1 shows total energy intake per day and some descriptive parameters. Oral intake was measured by 72-hour dietary recall and analyzed by Dietsource 3.0, Novartis Medical Nutrition.

Parenteral support was patient tailored and carefully monitored by the Nutrition Unit. PN was infused 3 to 7 times per week, depending on the patient’s chronic or transient intestinal failure, and was adjusted to individual needs and to their degree of intestinal failure. In aSBS HPN patients, the average PN composition was: lipids (20 g/day), amino acids solution (36 g/day) and glucose (125 g/day). In nSBS patients, the PN regime was: lipids (25 g/day), amino acids solution (50 g/day) and glucose (155 g/day). Electrolytes, minerals, vitamins and trace elements were given in sufficient amounts to maintain normal blood concentrations.

### Plasma sampling

In all subjects, blood samples were obtained after overnight fasting. In patients under PN, intravenous nutrition was stopped at least 8 hours before blood sampling. Blood was collected in disodium-EDTA and immediately centrifuged at 2,000 x*g* at 4°C for 30 min. Clear, non-hemolyzed plasma was separated and kept at -80°C for Apo AIV and citrulline measurements.

### Determination of Apo AIV

**Fig 1:**
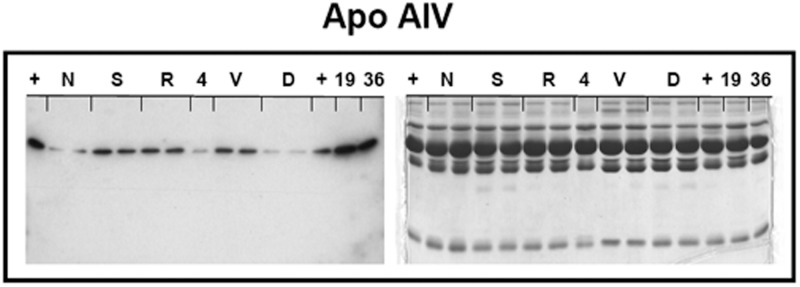
Representative Western blot of Apo AIV in human plasma of SBS patients and healthy subjects. After blotting (left membrane), gel proteins were stained with Coomassie blue (right) to ensure that the same amount of diluted plasma (1/50) was loaded in each lane. Samples were processed per duplicate in SDS-PAGE gel (right). Five SBS patients (codes: ‘N’, ‘S’, ‘R’, ‘V’ and ‘D’) and three individuals from the control group (codes: ‘4’, ‘19’ and ‘36’) were analyzed. Plasma (+): was used as internal control (100 AU).

Plasma Apo AIV was analyzed by Western blot (WB) assay [23, 31]. Proteins were separated by 10% SDS-PAGE (i.e., sodium dodecylsulfate-polyacrylamide gel electrophoresis) and transferred into "Immovilon-P" membranes (Millipore^®^, Bedford, USA) (Fig 1). The polyclonal anti-human Apo AIV IgG, developed at our laboratory and obtained from rabbit, was used at 1/500 dilution as the primary antibody. As a secondary antibody, we used anti-IgG from a commercial rabbit kit obtained in pig (marked with horseradish peroxidase -HRP) at 1/15,000 dilution (Dako^®^, Glostrup, Denmark). Finally, non-specific binding was blocked with bovine serum albumin (BSA) at 2 g/100 mL in PBS, pH = 7.4. The WB was developed using the SuperSignal West Pico chemiluminescent substrate for HRP detection (Pierce^®^, Rockford, Illinois, USA). Relative amounts were estimated by densitometry scanning (Phoretix 1D Gel Analysis Software, Nonlinear Dynamics, Newcastle, UK). For each SDS-PAGE gel, plasma from a young healthy subject with 13.1 mg/100 mL was used as an internal control and defined as 100 AU. Values were measured in arbitrary units (AU). These AU values were then converted to concentrations (mg/100 mL). All samples were processed twice in two separate gels.

### Determination of citrulline

Plasma citrulline analysis was performed at the Division of Inborn Errors of Metabolism-IBC (Hospital Clínic Barcelona, Spain) by high performance liquid chromatography (HPLC) using a reversed-phase column (Pico-Tag, WatersCorporation). About 100 μL of plasma were mixed with 400 μM of homoarginine, which was used as an internal standard. This mixture was then filtrated through an Amicon Ultra 0.5 mL, 10,000 MW cut-off filter (Millipore Iberica, Madrid, Spain) and derivatized using phenylisothiocyanate [32]. The product (10 μL) was injected into an HPLC system (Alliance 2690, Waters Corporation, Milford, USA). Gradient elution and detection were carried out at 254 nm with a Waters 486 detector (Waters Corporation). Plasma citrulline was quantified based on comparison with a standard containing a mix of the main amino acids (acid, neutral and basic). Data were processed with Empower software (Waters Corporation).

### Statistical analysis

Statistical analyses were performed at Mixestat S.L. Summary statistics were used to describe populations, mean and 95% confidence intervals, standard deviations and medians for continuous variables and counts and percentages for categorical variables. Testing normality for variables Apo AIV, citrulline and RSBL was checked with Shapiro-Wilk or Kolmogorov-Smirnoff test. Given normality, the linear relationship between variables was estimated with Pearson correlation, applying Fisher’s transformation to derive its confidence limits and a p-value under specified null hypotheses. Bootstrap resampling Spearman correlation (r_b_) was applied to analyze subgroups with fewer than 10 patients. An exploratory receiver operating characteristic (ROC) curve as well as the corresponding area under the curve (AUC) was fitted using SAS program of Schneider [33]. The citrulline value of 20 μM defined as a cut-off point to discriminate between transient and chronic intestinal failure after Crenn et al. [19], was used as gold standard. Both, data management and statistics were performed with SAS^®^ 9.4 software.

## Results

Table 1 shows summary statistics for some parameters and the energy intake of all SBS patients (S1 Table). RSBL (range 15–49 cm) was lower in seven patients with chronic intestinal failure who belonged to aSBS HPN group. In the remaining patients, RSBL fell into three distinct intervals: 50–99 cm (34%), 100–149 cm (28%) and 150–200 cm (19%). Neither Apo AIV nor citrulline showed a correlation with daily oral energy intake, age or body weight (data not shown).

**Fig 2:**
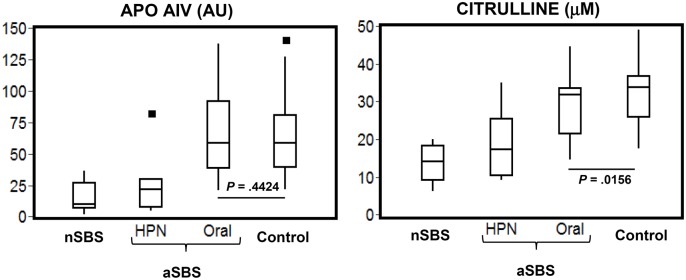
Box plots for plasma concentrations of Apo AIV and citrulline in selected SBS patients and healthy subjects. SBS groups: non-adapted nSBS (n = 8), adapted SBS HPN group (n = 7), and adapted SBS ORAL group (n = 19). Healthy subjects -Control group (n = 39). Outliers shown as black squares. *P<* .05 between aSBS ORAL and Control groups.

Apo AIV concentrations, mg/mL mean values, revealed two broad clusters of SBS patients (Fig 2): for the first cluster (n = 15) these were 16 AU (2.1 mg/100 mL) for the nSBS group and 24 AU (3.2 mg/100 mL) for the aSBS HPN group, and for the second cluster (n = 19) were 66 AU (8.7 mg/100 mL) for the aSBS ORAL group. Differences between clusters were statistically significant, (95% CI: 24, 65; *P* = .0001). In contrast, the distribution of citrulline concentrations showed no clusters but a distinct mean for each group (Fig 2): 14.8 μM for the nSBS group, 19.0 μM for the aSBS HPN group, 29.6 μM for the aSBS ORAL group and 31.9 μM for the control group (S2 Table).

**Fig 3:**
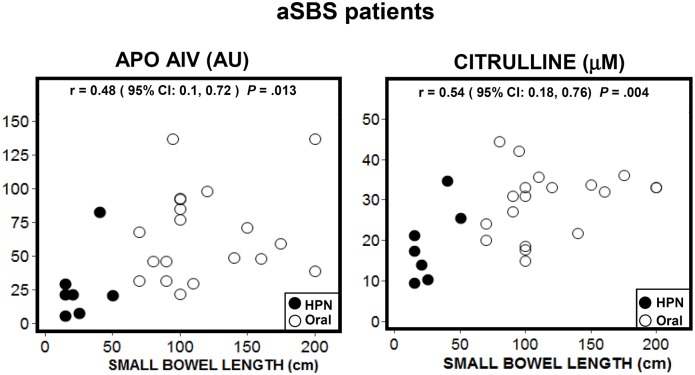
Scatter plots for Apo AIV and citrulline plasma values with the RSBL in all adapted SBS patients. (●) HPN group (n = 7) and (o) ORAL group (n = 19).

Apo AIV and citrulline correlated positively and significantly with RSBL as shown in Fig 3. However, this correlation vanished when only patients above the 50 cm threshold for RSBL were included: Apo AIV (r = 0.24, CI: −0.22, 0.62, *P* = .2896) and citrulline (r = 0.26, CI: −0.21, 0.63, *P* = .2634). Further, aSBS patients (HPN and ORAL groups) were split into two subgroups according to the lack (n = 16) or presence (n = 10) of ileum. Difference between the mean values of RSBL of these subgroups (100 cm and 83 cm, respectively) was not significant (95% CI: −28.9, 62.9, *P =* .4542). Only citrulline showed statistical significance between the mean values of these subgroups: 30.7 μM (n = 16) versus 20.4 μM (n = 10) (95% CI: 3.43, 17.04, *P =* .0048).

**Fig 4:**
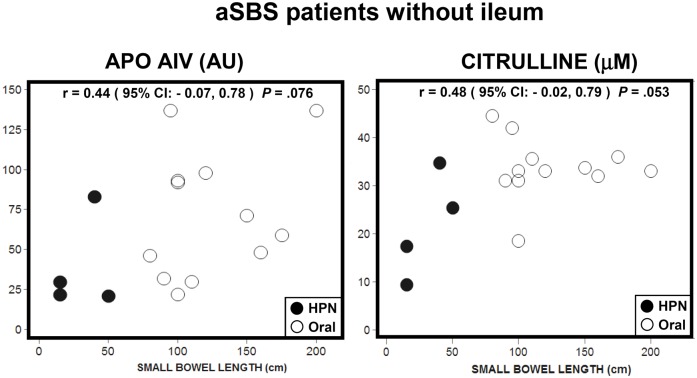
Scatter plots for Apo AIV and citrulline plasma values with the RSBL in adapted SBS patients without ileum. (●) HPN group (n = 4) and (o) ORAL group (n = 12).

**Fig 5:**
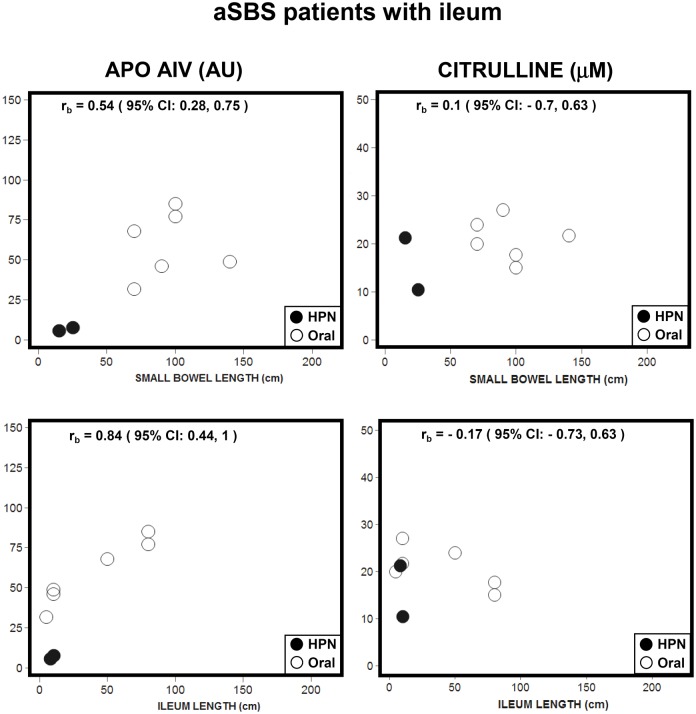
Scatter plots for Apo AIV and citrulline plasma values with the RSBL and a remnant ileum length in adapted SBS patients with ileum and proximal part. (●) HPN group (n = 2) and (o) ORAL group (n = 6). Bootstrap resampling correlation was performed.

Figs 4 and 5 show Apo AIV and citrulline values plotted against total RSBL for aSBS patients categorized by type of anastomosis: jejunocolic and jejunostomy (Fig 4, without ileum and proximal part, n = 16) and jejunoileocolic (Fig 5, with ileum and proximal part, n = 8; two patients without proximal part were not considered). Apo AIV and citrulline in aSBS patients without ileum (Fig 4)) showed no correlation with RSBL. In aSBS patients with ileum (Fig 5), bootstrap resampling was used to analyze the data. Apo AIV concentrations showed a significant correlation with RSBL and with ileum remnant length. In contrast, citrulline concentrations showed no correlation, i.e., neither with RSBL nor with ileum remnant length. Thus, Apo AIV values in patients with comparable ileum length are not alike when the length of their proximal intestine is different (lower part of Fig 5).

**Fig 6:**
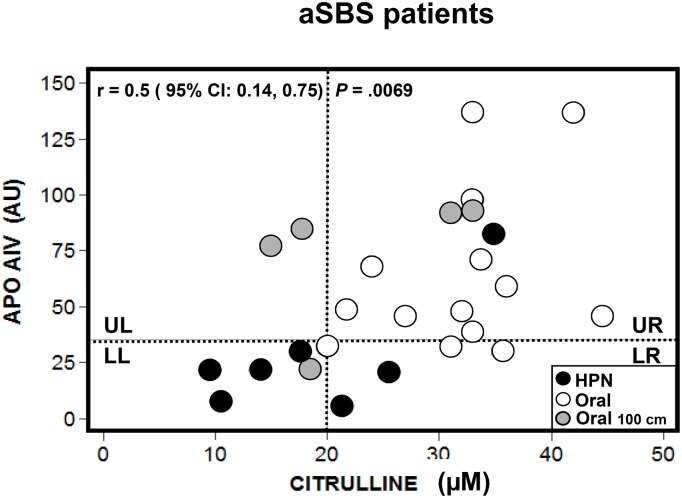
Scatter plot for Apo AIV with citrulline plasma values in all adapted SBS patients. (●) HPN group (n = 7) and (o) ORAL group (n = 19). ROC analysis: tentative cut-off point of 32 AU (4.6 mg/100 mL) for Apo AIV (20 μM citrulline as gold standard). Grey circles show patients (‘G’, ‘H’, ‘Q’, ‘R’ and ‘S’) with similar RSBL (100 cm).

Table 2 (S1 Table) summarizes the individual data of RSBL and plasma markers on aSBS patients. Patients were classified according to anastomosis type and nutritional group. Patients with RSBL >50 cm plus colon (partial or total) were all PN-independent. Based on individual citrulline and Apo AIV plasma levels, patients were distributed into the four quadrants shown in Fig 6.

**Table 2:** Plasma markers and short bowel anatomy of all adapted SBS patients (aSBS).

Anastomosis	Code	aSBS	RSBL (cm)				Apo AIV(AU)	Citrulline (μM)	Quadrant Fig 6
Type	Patient	Group	Total	Proximal	Ileum	% Ileum/Total			
Type III	A	HPN	15	7	8	53	6	21.3	LR
	B	HPN	20	0	20	100	22	14.0	LL
	C	HPN	25	15	10	40	8	10.5	LL
	D	ORAL	70	65	5	7	32	20.0	LL-LR
	E	ORAL	70	20	50	71	68	24.0	UR
	F	ORAL	90	80	10	11	46	27.0	UR
	G	ORAL	100	20	80	80	85	17.7	UL
	H	ORAL	100	20	80	80	77	15.0	UL
	I	ORAL	140	130	10	7	49	21.7	UR
	J	ORAL	200	0	200	100	39	33.0	UR
Type II	K	HPN	15	15	-	-	30	17.5	LL
	L	HPN	15	15	-	-	22	9.5	LL
	M	HPN	40	40	-	-	83	34.8	UR
	N	HPN	50	50	-	-	21	25.5	LR
	O	ORAL	80	80	-	-	46	44.5	UR
	P	ORAL	95	95	-	-	137	42.0	UR
	Q	ORAL	100	100	-	-	22	18.5	LL
	R	ORAL	100	100	-	-	92	31.0	UR
	S	ORAL	100	100	-	-	93	33.0	UR
	T	ORAL	110	110	-	-	30	35.6	LR
	U	ORAL	150	150	-	-	71	33.7	UR
	V	ORAL	160	160	-	-	48	32.0	UR
	W	ORAL	200	200	-	-	137	33.0	UR
Type I	X	ORAL	90	90	-	-	32	31.0	LR-UR
	Y	ORAL	120	120	-	-	98	33.0	UR
	Z	ORAL	175	175	-	-	59	36.0	UR

Remnant small bowel length (RSBL). Ileum length from patients with anastomosis Type I and Type II was not available (-). Quadrants defined in Fig 6: UL Upper Left, LL Lower Left, UR Upper Right, LR Lower Right.

Apo AIV and citrulline plasma concentrations in all aSBS patients are plotted in Fig 6. Both biomarkers were positively correlated (r = 0.5; 95% CI = 0.14, 0.75; *P* = .0069). The ROC curve analysis yields a sensitivity of 75% a specificity of 72%, a positive predictive value of 60% and a negative predictive value of 87.5%. The AUC has an estimate of 0.74 at a 95% CI, range: 0.52 to 0.95. The tentative cut-off point for Apo AIV, the threshold that discriminates between aSBS on HPN from the aSBS ORAL group and controls as shown in Fig 6, is around 32 AU (4.6 mg /100 mL). This value should be taken as tentative because of both the exploratory nature of the analysis and the moderate accuracy. However, this is consistent with the results on Fig 2, where a value of Apo AIV around 30 AU splits patients between aSBS on HPN and aSBS ORAL groups.

Accordingly, the Apo AIV (32 AU) and citrulline (20 μM) threshold values are plotted with dotted lines. These threshold lines divide the Fig 6 into four quadrants: upper left (UL), high Apo AIV, low citrulline; upper right (UR), high Apo AIV and citrulline; lower left (LL), low Apo AIV and citrulline; and lower right (LR), low Apo AIV and high citrulline. The majority of patients under HPN (n = 4) fell into the LL quadrant and had a RSBL under 25 cm. Five aSBS ORAL patients with the same RSBL (100 cm, marked in grey circles) fell into different quadrants: patients ‘G’ and ‘H’ in UL; patient ‘Q’ in LL and patients ‘R’ and ‘S’ in UR.

Finally, the Apo AIV cut-off value (32 AU) performs better than citrulline to pinpoint PN weaning since, within the aSBS HPN group, it sets apart 6 out of 7 patients in need of PN. This is slightly better than citrulline that only sets apart 4 out of 7.

**Fig 7:**
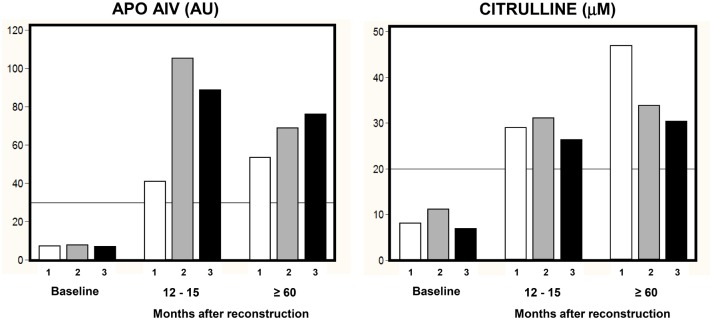
Process of intestinal adaptation in nSBS patients. Bar diagrams for the evolution of Apo AIV and citrulline plasma values in three non-adapted SBS patients ‘1’, ‘2’ and ‘3’. RSBL: 60 cm without ileum ‘1’, 70 cm ‘2’ and 150 cm ‘3’. Measurements were performed on the same individual: before (baseline) and after surgery at 12–15 and at ≥ 60 months (full adaptation). Lines show the threshold values as described on Fig 6.

The process of intestinal adaptation was monitored in three selected nSBS patients with different RSBL and intestinal anastomoses (Fig 7). Their clinical parameters improved and the biomarkers’ values increased with time in all patients reflecting the process of intestinal adaptation. Plasma Apo AIV concentrations on patient ‘1’ (without ileum) were low and increased gradually, and citrulline showed a steady increase, peaking at the time of full adaptation, approximately 5 years. Patients ‘2’ and ‘3’ showed a similar evolution in both biomarkers values.

## Discussion

In humans, Apo IV and citrulline are both synthesized almost exclusively in the enterocytes of the small intestine. Thus, they have been proposed as biomarkers to monitor intestinal function [19, 23, 34]. SBS patients have suffered massive small bowel resection, which is a cause of intestinal failure. Consequently, they undergo a process of adaptation that restores the intestinal function, a process that may last up to two years. Thereafter, patients are considered to be adapted [6]. However, in some of them the absorptive capacity may still keep on improving for several years following resection [7]

Apo AIV concentrations in nSBS and aSBS HPN patients were clearly distinct from those of oral aSBS patients and controls. The exploratory ROC analysis indicated that a cut-off line divided the two sorts of patients at approximately 32 AU (4.6 mg/ mL). This is important because it may signal the threshold that discriminated between patients who need PN care and those who do not. Moreover, it suggests that patients with Apo AIV plasma concentrations higher than 4.6 mg/mL had adapted and were therefore good candidates for PN weaning. However promising, since it does better than citrulline to pinpoint patients that may need PN care (Fig 6), this value should be regarded as tentative because patient numbers are too small to form definite conclusions.

In oral aSBS patients, Apo AIV and citrulline concentrations overlapped with those of the controls. The coincidence between the groups was higher for Apo AIV than for citrulline. Peters et al. [3] showed that adapted oral SBS patients with RSBL between 80 cm and 110 cm had average citrulline levels similar to those of healthy subjects (32 μM vs 34 μM, respectively). Therefore, these results endorse Apo AIV as a biomarker.

Because citrulline is synthesized in the small bowel, plasma citrulline concentrations in SBS patients have been tentatively associated with absorptive function and, accordingly, with RSBL [3, 18, 35, 36]. We found that both Apo AIV and citrulline values were correlated with RSBL. The correlation, however, disappeared when we excluded patients with RSBL <50 cm. In the presence of the colon, a 50 cm to 100 cm RSBL has been identified as the interval above which patients can be easily weaned off PN [2, 3]. Thus, it is important to establish whether the biomarkers’ values correlate with RSBL. Crenn et al. [18] found a high correlation between plasma citrulline concentrations and RSBL. Their study included patients with transient and chronic intestinal failure. Luo et al. [24] reported similar findings, albeit with a much lower correlation. However, Peters et al. [3] challenged such results. They found that citrulline concentrations in patients with celiac disease, refractory celiac disease and SBS did not correlate with RSBL. Thus, they concluded that single citrulline plasma concentration values cannot measure enterocyte absorption capacity and function. Moreover, they also noted that the strong correlation between citrulline and small bowel length found by Crenn et al. [18] could be related to a dataset with a large proportion of patients with a RSBL <50 cm. Similarly, Peters et al. [27] suggested that the correlation measured by Luo et al. [24] was enhanced by the uneven distribution of patients in their dataset: 19 patients with SB <200 cm and 3 patients with SB >300 cm. Overall, biomarkers’ values appeared to be influenced by factors other than RSBL and absorption.

Apo AIV and citrulline have preferred sites of expression in the small bowel. While Apo AIV is mainly synthesized in the ileum [16, 21], citrulline is mainly synthesized in the proximal intestine [10, 28]. Thus, we hypothesized that the anatomy of the RSBL should be taken into consideration in the interpretation of the results. In patients without ileum, Apo AIV and citrulline displayed a weaker correlation with RSBL. Furthermore, Apo AIV values showed a high dispersion. Citrulline values from aSBS ORAL patients with RSBL >100 cm levelled out at approximately 34 μM (Fig 3). The shape of this distribution agreed with the findings of other studies [25], showing that the relationship between citrulline and short bowel length is quadratic, not linear. This implies that citrulline concentrations are not expected to increase with RSBL beyond a threshold—approximately 100cm in our study—which Papadia et al. [25] modeled to be 240 cm.

In patients with ileum, Apo AIV concentrations showed a high positive correlation with RSBL and an even higher correlation with ileum length. In contrast, no correlation was established with citrulline concentrations. Therefore, for this group of patients, Apo AIV could be more reliable than citrulline as a biomarker for adaptation. In aSBS ORAL patients (Table 2), high Apo AIV values (85 and 77 AU) and low citrulline values (17.7 and 15.0 μM) were found in patients with the largest percentage of RSBL being the ileum, i.e., 80% of a RSBL of 100 cm. On the contrary, high citrulline (ranging from 31.0 to 44.5 μM) values were found in patients who had only proximal intestine. For aSBS HPN patients, the biomarker values also reflected the short bowel anatomy. Patients with RSBL <25 cm displayed low Apo AIV (<32 AU) and citrulline (<20 μM) values. Patients with a short RSBL (15 to 50 cm) displayed Apo AIV values below the threshold of 32 AU (6 to 21 AU) and citrulline values close to the 20 μM threshold (21.3 to 25.5 μM). The low Apo AIV values in patients ‘A’ and ‘C’ can be explained by a shorter ileum and proximal part.

In addition to the aSBS HPN patients, the LL quadrant (Fig 6) also included an aSBS ORAL patient with a jejunocolic anastomoses and 100 cm RSBL; this position is unrepresentative of such patients due to the length of the remnant short bowel. While the lack of ileum may account for the low Apo AIV values (22 AU), the low citrulline values (18.5 μM) were too low for a patient ‘Q’ with 100 cm RSBL. These low values could be a reflection of villous atrophy related to the patient’s old age, i.e., 83 years, the oldest of our study. Jie ZG1 et al. [37] reported large differences between young and old humans in the structure of the mucosa that were related to age. They found that older patients had shorter and thicker villi and a thinner mucosa than younger ones. These differences may therefore account for the reduced citrulline production.

Five aSBS ORAL patients ‘G’, ‘H’, ‘Q’, ‘R’ and ‘S’ had the same RSBL of 100 cm. Two patients ‘G’ and ‘H’ fell into the UL quadrant. The large percentage of their ileum (80% from a RSBL of 100 cm) accounted for the high Apo AIV values (85 and 77 AU). The other two patients without ileum ‘R’ and ‘S’ fell into the UR quadrant; they presented high plasma biomarkers values (Apo AIV: 92 and 93 AU; citrulline: 31 and 33 μM), probably related to the improving adaptation of intestinal proximal part. Then, in all these patients the citrulline showed a different intestinal adaptation pattern. Only the oldest patient ‘Q’ also without ileum, fell into the LL quadrant like the majority of aSBS HPN patients with <50 cm of RSBL. These results imply that besides having biomarker values in the control range, the short bowel anatomy and the mucosa morphological changes are key in determining PN dependence.

Finally, the low values of Apo AIV in four patients who fell into the LR quadrant, two under HPN and two on oral-only regimen, could be linked either to a very short ileum (8 cm in patient ‘A’ under HPN) or a lack of ileum in patients ‘N’, ‘T’ and ‘X’. Therefore, presence of ileum and proportion of jejunum *vs* ileum in the RSBL has an influence on the Apo AIV.

Most aSBS HPN patients fell into the LL quadrant, but one stands as an exception to this pattern and fell into the UR quadrant. This patient ‘M’ had high Apo AIV (83 AU) and citrulline (34.8 μM) values and only a proximal 40cm RSBL. Although, such a RSBL is commonly associated with PN care, the citrulline value is not [3, 35]. Thus, apart from anatomy and RSBL, other non intestinal factors may also play an influential role in determining Apo AIV and citrulline concentrations. For instance, inflammation [25, 38] and renal failure [19] have been reported to raise citrulline concentrations. Additionally, high plasma citrulline concentrations have been reported in SBS hyperphagic patients [18]. This could explain high Apo AIV and citrulline concentrations in some SBS patients with a RSBL <50 cm.

To test this hypothesis we measured Apo AIV and citrulline concentrations in three patients (Fig 7) through their period of adaptation, one of whom, patient ‘1’, had declared hyperphagic behavior. The biomarker values in patient ‘1’ displayed a distinct pattern of evolution. Apo AIV increased slowly through all stages and remained the lowest in the cohort, as expected in patients without ileum. In contrast, citrulline increased steadily and showed the highest value among the three patients at the full adaptation stage, approximately 48 μM, which is 1.5 times higher than the average in the controls. This pattern could be explained by the interaction between adaptive processes triggered by resection and hyperphagia. Hyperphagia seems to develop as a result of ileum removal. The ileum emits satiety signals to the brain concerning several proteins: glucagon-like-peptide-1 (GLP-1), glucagon-like-peptide-2 (GLP-2), peptide-tyrosine-tyrosine (PYY), Apo AIV and enterostatin [16, 39, 40]. As a result, ileum resection is likely to alter patients’ appetite. After resection, the process of intestinal adaptation involves morphological and functional changes through which the jejunum acquires some functional qualities of the missing ileum, such as increased mRNA protein levels and subsequently stimulated production of GLP-1, GLP-2, PYY and Apo AIV [40, 41]. However, in patients with a missing ileum, GLP-1, GPL-2 and PYY cannot be expressed; only Apo AIV, which has a secondary site of expression in the proximal jejunum, can be synthesized. Thus, lack of ileum may account for a low Apo AIV. Supplying patients with Teduglutide, a recombinant analog of GLP-2, can restore intestinal function by inducing morphological mucosa changes in the RSBL [42] thereby reducing PN dependence [11].

The SBS adapts to hyperphagia through morphological and structural changes. For instance, in a murine SBS model [43], researchers described increased intestinal and mucosal weights. These are indicative of an amplified absorptive surface with increased mucosal folds and villous thickness. In humans with SBS, morphological changes triggered by hyperphagia are part of the adaptative process because they ultimately lead to increased intestinal absorption [44]. The high citrulline concentrations found in patient ‘1’, are expected, since higher plasma citrulline values have been reported in hyperphagic patients over normophagic ones [18]. Hyperphagia in SBS patients was asserted to stimulate intestinal adaptation [44]. Thus, hyperphagia in patient ‘1’ probably led to an increased rate of adaptation as well as a shorter PN dependence.

In conclusion, concomitant measurements of Apo AIV and citrulline in SBS patients show that both biomarkers complement each other in monitoring the process of intestinal morphological changes after resection. This is because by having a preferred site of expression in the RSBL, both the length and the anatomy of the RSBL (remnant ileum, mucosa growth, villi thickness) strongly influence both biomarkers. The Apo AIV cut-off value, discriminating between patients who need PN and those who do not, is set at 4.6 mg/100 mL. This value is tentative, owing to the small number of patients, and requires further research. Consequently, monitoring and managing the SBS intestinal adaptation process can be improved by acknowledging factors that may influence the biomarkers’ values, including the RSBL, hyperphagia and old age.

## Supporting Information

S1 Table Short Bowel Syndrome (SBS) patients’ data. (PDF)

S2 Table Control group data. (PDF)
