# Supplementary material for: Apo AIV and Citrulline Plasma Concentrations in Short Bowel Syndrome Patients: The Influence of Short Bowel Anatomy
Source: PLoS One. 2016 Sep 30;11(9):e0163762. doi: 10.1371/journal.pone.0163762 (PMC5045203; doi:10.1371/journal.pone.0163762)
Supplement: S1 Table — (PDF) [file pone.0163762.s001.pdf]

S1 Table. Short Bowel Syndrome (SBS) patient's data

Code SBS Patient	SBS Group		Characteristics			Remnant Short Bowel Length			Etiology	Anastomoses	Energy intake (MJ / day)			Plasma biomarkers	
Table 2	Adapted	Nutritional regimen	Age (years)	Sex	Weight (Kg)	Total (cm)	Ileum (cm)	Colon	resection	Type	Total	Oral	Parenteral	ApoAIV (AU)	Citrulline (µM)
A	yes	HPN	26	women	41,3	15	8	yes	ischemic	III	15,5	11,6	3,9	6	21,3
B	yes	HPN	43	women	57,8	20	20	yes	ischemic	III	12,4	9,7	2,7	22	14,0
M	yes	HPN	59	men	79,5	40	0	no	ischemic	II	14,0	11,5	2,5	83	34,8
N	yes	HPN	32	men	62,0	50	0	no	ischemic	II	17,2	14,7	2,6	21	25,5
K	yes	HPN	28	men	50,5	15	0	no	ischemic	II	18,5	14,6	3,9	30	17,5
L	yes	HPN	61	men	63,5	15	0	no	ischemic	II	16,2	11,4	4,8	22	9,5
C	yes	HPN	64	women	50,0	25	10	no	ischemic	III	12,9	9,0	3,9	8	10,5
G	yes	Oral	32	men	94,0	100	80	yes	ischemic	III	12,6	12,6	0	85	17,7
Z	yes	Oral	46	women	53,0	175	0	no	actinic	I	11,0	11,0	0	59	36,0
T	yes	Oral	75	men	60,0	110	0	no	ischemic	II	8,2	8,2	0	30	35,6
O	yes	Oral	41	women	49,2	80	0	no	actinic	II	12,5	12,5	0	46	44,5
E	yes	Oral	65	women	69,0	70	50	no	ischemic	III	8,2	8,2	0	68	24,0
F	yes	Oral	69	women	92,0	90	10	yes	ischemic	III	10,3	10,3	0	46	27,0
U	yes	Oral	68	men	64,7	150	0	no	actinic	II	12,8	12,8	0	71	33,7
I	yes	Oral	55	women	44,3	140	10	yes	actinic	III	10,1	10,1	0	49	21,7
R	yes	Oral	72	women	75,0	100	0	no	actinic	II	6,6	6,6	0	92	31,0
S	yes	Oral	54	women	56,2	100	0	no	ischemic	II	9,6	9,6	0	93	33,0
J	yes	Oral	64	women	60,0	200	200	yes	actinic	III	9,3	9,3	0	39	33,0
V	yes	Oral	63	women	59,7	160	0	no	actinic	II	12,2	12,2	0	48	32,0
D	yes	Oral	67	men	57,4	70	5	yes	ischemic	III	13,1	13,1	0	32	20,0
X	yes	Oral	69	men	49,3	90	0	no	actinic	I	13,0	13,0	0	32	31,0
P	yes	Oral	77	women	60,0	95	0	no	ischemic	II	10,9	10,9	0	137	42,0
Q	yes	Oral	83	women	63,0	100	0	no	actinic	II	7,9	7,9	0	22	18,5
W	yes	Oral	53	women	58,0	200	0	no	actinic	II	11,6	11,6	0	137	33,0
Y	yes	Oral	55	men	46,5	120	0	no	actinic	I	14,9	14,9	0	98	33,0
H	yes	Oral	33	men	90,4	100	80	yes	ischemic	III	12,6	12,6	0	77	15,0
2	non-adapted	HPN	44	men	60,2	70	25	no	ischemic	I	10,5	7,8	2,6	8	11,0
undetermined	non-adapted	Oral	73	women	49,0	120	no determined	no	actinic	I	10,5	10,5	0	3	20,0
undetermined	non-adapted	Oral	33	men	61,8	90	75	no	ischemic	I	10,5	10,5	0	20	17,0
undetermined	non-adapted	HPN	60	women	41,0	50	0	no	actinic	I	10,5	6,1	4,4	35	16,0
3	non-adapted	Oral	48	women	46,0	150	50	no	ischemic	III	11,3	11,3	0	7	6,7
1	non-adapted	HPN	43	men	83,0	60	0	no	ischemic	I	10,5	9,6	3,2	7	7,9
undetermined	non-adapted	Oral	59	men	78,6	290	90	yes	ischemic	III	12,0	12,0	0	12	12,6
undetermined	non-adapted	HPN	60	women	54,0	50	no determined	no	actinic	I	12,4	9,3	3,1	36	20,0
