# Supplementary material for: Apo AIV and Citrulline Plasma Concentrations in Short Bowel Syndrome Patients: The Influence of Short Bowel Anatomy
Source: PLoS One. 2016 Sep 30;11(9):e0163762. doi: 10.1371/journal.pone.0163762 (PMC5045203; doi:10.1371/journal.pone.0163762)
Supplement: S2 Table — (PDF) [file pone.0163762.s002.pdf]

S2 Table. Control group data

CONTROL Group			Plasma biomarkers	
**Code**	**Age (years)**	**Sex**	**ApoAIV (AU)**	**Citrulline (μM)**
**1**	56	women	56	36
**2**	64	women	37	30
**3**	77	women	31	39
**4**	37	men	30	37
**5**	30	women	27	22
**6**	32	women	44	24
**7**	44	men	77	27
**8**	25	men	83	35
**9**	55	men	39	49
**10**	24	women	40	29
**11**	63	women	72	48
**12**	52	women	28	27
**13**	31	men	102	29
**14**	54	men	43	39
**15**	39	women	91	34
**16**	27	men	101	37
**17**	46	women	70	38
**18**	55	women	56	26
**19**	58	men	146	37
**20**	43	women	59	34
**21**	64	men	40	34
**22**	63	women	95	36
**23**	78	women	98	37
**24**	79	men	30	33
**25**	70	men	77	24
**26**	72	men	60	35
**27**	71	men	61	35
**28**	78	women	60	41
**29**	54	men	43	27
**30**	51	women	23	23
**31**	58	men	28	36
**32**	55	men	43	39
**33**	57	men	81	18
**34**	25	women	95	21
**35**	32	women	66	19
**36**	28	men	127	32
**37**	27	men	51	29
**38**	40	men	50	25
**39**	28	women	64	24
